# Iron Delivery through Membrane Vesicles in Corynebacterium glutamicum

**DOI:** 10.1128/spectrum.01222-23

**Published:** 2023-05-08

**Authors:** Kayuki Kawashima, Toshiki Nagakubo, Nobuhiko Nomura, Masanori Toyofuku

**Affiliations:** a Graduate School of Life and Environmental Sciences, University of Tsukuba, Tsukuba, Ibaraki, Japan; b Faculty of Life and Environmental Sciences, University of Tsukuba, Tsukuba, Ibaraki, Japan; c Microbiology Research Center for Sustainability (MiCS), University of Tsukuba, Tsukuba, Ibaraki, Japan; University of Minnesota Twin Cities

**Keywords:** membrane vesicles, mycolic acid-containing bacteria, iron acquisition

## Abstract

Bacterial cells form and release membrane vesicles (MVs) originating from cellular membranes. In recent years, many biological functions of bacterial MVs have been identified. Here, we show that MVs derived from Corynebacterium glutamicum, a model organism for mycolic acid-containing bacteria, can mediate iron acquisition and other phylogenetically related bacteria. Lipid/protein analysis and iron quantification assay indicate that C. glutamicum MVs formed by outer mycomembrane blebbing can load ferric iron (Fe^3+^) as its cargo. Iron-loaded C. glutamicum MVs promoted the growth of producer bacteria in iron-limited liquid media. MVs were received by C. glutamicum cells, suggesting a direct transfer of iron to the recipient cells. Cross-feeding of C. glutamicum MVs with phylogenetically close (Mycobacterium smegmatis and Rhodococcus erythropolis) or distant (Bacillus subtilis) bacteria indicated that C. glutamicum MVs could be received by the different species tested, while iron uptake is limited to M. smegmatis and R. erythropolis. In addition, our results indicate that iron loading on MVs in C. glutamicum does not depend on membrane-associated proteins or siderophores, which is different from what has been shown in other mycobacterial species. Our findings illustrate the biological importance of MV-associated extracellular iron for C. glutamicum growth and suggest its ecological impact on selected members of microbial communities.

**IMPORTANCE** Iron is an essential element of life. Many bacteria have developed iron acquisition systems, such as siderophores, for external iron uptake. Corynebacterium glutamicum, a soil bacterium known for its potential for industrial applications, was shown to lack the ability to produce extracellular, low-molecular-weight iron carriers, and it remains elusive how this bacterium acquires iron. Here, we demonstrated that MVs released from C. glutamicum cells could act as extracellular iron carriers that mediate iron uptake. Although MV-associated proteins or siderophores have been shown to play critical roles in MV-mediated iron uptake by other mycobacterial species, the iron delivery through C. glutamicum MVs is not dependent on these factors. Moreover, our results suggest that there is an unidentified mechanism that determines the species specificity of MV-mediated iron acquisition. Our results further demonstrated the important role of MV-associated iron.

## INTRODUCTION

Iron is an essential element for virtually all living organisms. In most cases, organisms acquire external iron in heme-bound or non-heme forms. In bacteria, siderophores and low-molecular-weight chelators for ferric iron are commonly utilized for bacterial uptake of external non-heme iron. Once siderophores are secreted into the extracellular milieu, they chelate ferric iron, which is imported into bacterial cells by specific uptake systems (1). While siderophores play crucial roles in bacterial physiology and ecology, some bacteria, such as Corynebacterium glutamicum, cannot produce siderophores, and iron acquisition systems remain largely elusive.

Bacterial membrane vesicles (MVs) are nano-sized spherical structures consisting of lipid bilayers originating from the bacterial cellular membranes (2–5). MVs often carry proteins, nucleic acids, and other biomolecules involved in diverse biological processes (6). Recent studies have identified a new role for MVs in iron transport. In Pseudomonas aeruginosa, a protein TseF secreted through a type VI secretion system, a cell envelope-spanning secretory nanomachine, interacts with iron-binding Pseudomonas quinolone signal (PQS) that is associated with MVs released by P. aeruginosa (7). These MVs containing TseF and iron-binding PQS may facilitate iron uptake *via* direct interactions between TseF and both the Fe(III)-pyochelin receptor and porin OprF (7). Furthermore, Mycobacterium MVs containing siderophores have been reported to capture iron and mediate its uptake by Mycobacterium cells (8–11). These reports suggest that MVs are widely involved in iron transport, but only a limited number of studies have been conducted.

We recently reported that C. glutamicum, a model of mycolic acid-containing bacteria with a unique cell envelope structure, including the outer mycomembrane rich in highly hydrophobic mycolic acids and inner phospholipid membrane, forms MVs *via* different routes (12). Under normal culture conditions, C. glutamicum cells predominantly release mycolic acid-rich MVs, which are presumably formed by outer mycomembrane blebbing (12). Although mycomembrane-derived MVs (mMVs) are unique in their biochemical composition, physiological consequences of mMVs formation remain poorly understood.

In this study, we show that mMVs of C. glutamicum can load ferric iron and mediate iron acquisition. MVs loaded with iron promoted the growth of C. glutamicum under iron-limiting conditions. These results suggest that MVs allow this bacterium lacking typical siderophores to gather external iron. Furthermore, we provide evidence of the species specificity of iron acquisition mediated by C. glutamicum MVs. These results provide further evidence of the biological importance of MV-associated iron in bacteria.

## RESULTS

### Corynebacterium glutamicum MVs can load ferric iron.

**FIG 1:**
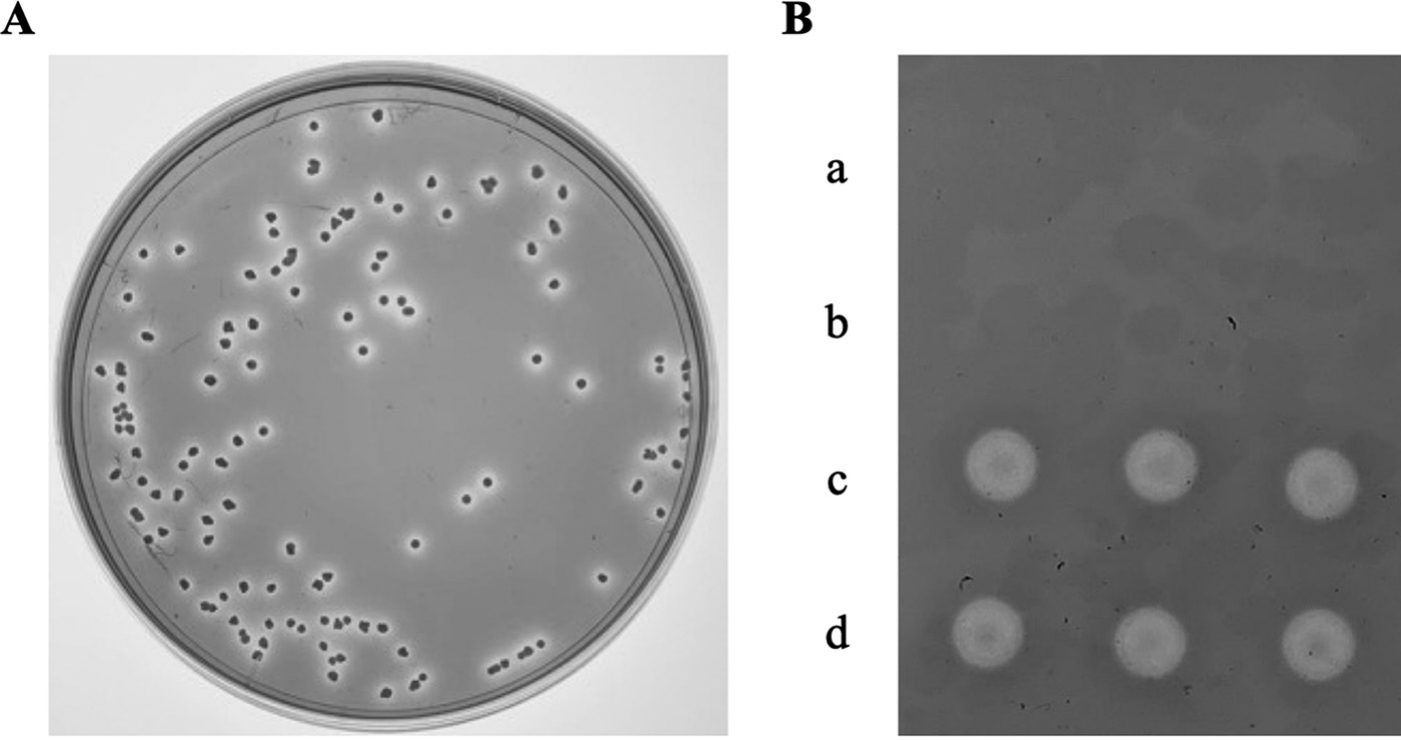
C. glutamicum release an iron chelator. (A) CAS assays of C. glutamicum cells were performed. Colonies were grown on LB agar plates and then layered with CAS agar. (B) MVs isolated from the culture supernatant have ferric iron chelate activity. Culture supernatant (a), ultracentrifugation supernatant (b), ultracentrifugation pellet (140 times more concentrated) (c), and purified MVs (140 times more concentrated) (d) were spotted onto an agar plate containing detection reagents in triplicate.

**FIG 2:**
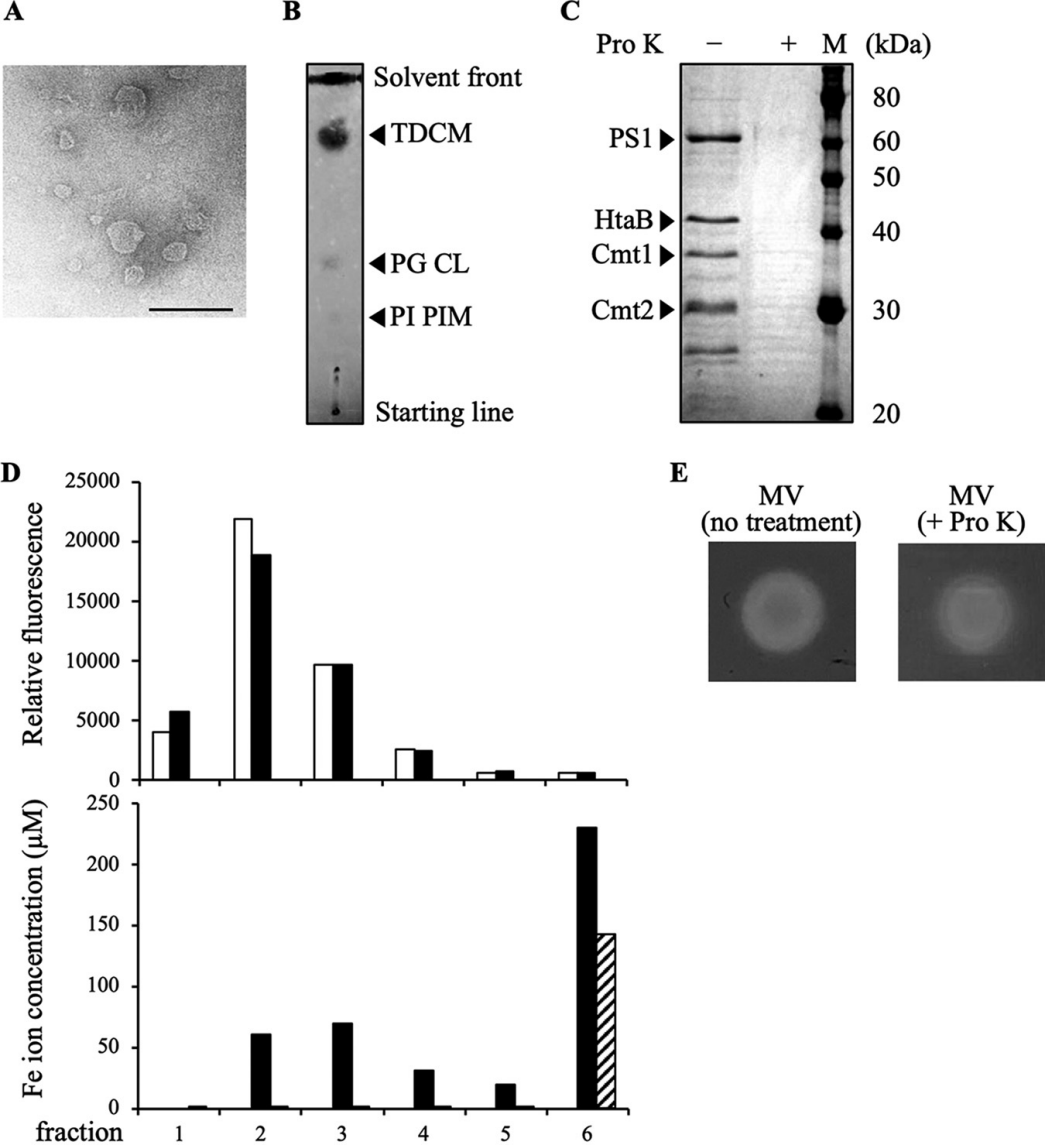
MVs derived from C. glutamicum mycomembrane can load ferric iron. (A) Transmission electron microscopic (TEM) images of MVs released by C. glutamicum in -Fe medium. Scale bar, 100 nm. (B) Major MV lipids were detected by thin-layer chromatography. TDCM, trehalose dicorynomycolic acid. PG, phosphatidylglycerol. CL, cardiolipin. PI, phosphatidylinnositol. PIM, phosphatidylinnositol mannoside. These lipids were separated using chloroform: methanol: H_2_O = 65:25:4 (vol/vol) as a solvent. All lipids were labeled with primuline and detected at 365 nm. (C) SDS-PAGE profiles of MV proteins are shown. 5 μg of MV proteins were applied. An equivalent amount of MVs that were proteinase K-treated were loaded onto the middle lane. M, marker proteins. PS1, corynomycoloyltransferase C chain A; HtaB, heme binding membrane protein; Cmt1 and Cmt2, trehalose corynomycolyltransferases. (D) MVs isolated from -Fe medium were mixed with ferric ion. After ultracentrifugation, MVs were fractionated by density gradient centrifugation. +Fe MV and -Fe MV indicate iron-loaded MVs and iron-free MVs. The MV content of each fraction was quantified using FM1-43 FX dye (white bar, -Fe MV; black bar, +Fe MV) (top). The iron ion concentration of each fraction was quantified by the ferrozine method (black bar, +Fe MV; shaded bar, FeCl_3_· 6H_2_O only) (bottom). (E) CAS assay of C. glutamicum MVs. Non-treated MVs (left), proteinase K-treated MVs (right) were spotted onto an agar plate containing detection reagents.

Corynebacterium glutamicum was reported not to produce siderophores (13–15). However, we observed a clear zone formed around each C. glutamicum ATCC13032 colony on a CAS assay plate, a dye-containing solid medium generally used to detect the iron-chelating activity of the samples (Fig. 1A). This result suggests the release of an iron chelator from C. glutamicum cells. Consequently, we collected the culture supernatant from the liquid C. glutamicum culture and performed the CAS assay to detect the iron chelating activity of the putative extracellular iron chelator(s). While there was no detectable activity in the culture supernatant, we detected iron chelating activity in the ultracentrifugation pellets of the culture supernatant of C. glutamicum grown in a synthetic medium in which iron was not added (-Fe medium) (Fig. 1B). Because C. glutamicum MVs have been previously found to be pelleted using ultracentrifugation (12), we assumed that MVs, at least in part, accounted for the iron chelating activity of the pellets. We thus purified MVs from the pellets by density gradient ultracentrifugation and found that the purified MVs also exhibited iron chelating activity (Fig. 1B). Transmission electron microscopy (TEM) images showed spherical lipid-bilayer structures under culture conditions similar to those previously reported for C. glutamicum MVs (12) (Fig. 2A). Thin-layer chromatography showed a high content of corynomycolic acid ester, suggesting that these MVs are primarily mycomembrane vesicles originating from the mycomembrane of C. glutamicum (12) (Fig. 2B). SDS-PAGE analysis of purified MVs revealed distinct bands (Fig. 2C). MVs (-Fe MVs) were mixed with ferric iron (+Fe MVs) and purified by two-step ultracentrifugation/density gradient ultracentrifugation as described under Materials and Methods to examine whether MVs can associate with iron (Fig. S1AB). The purified MVs were washed with 10 mM HEPES-NaOH buffer (pH 8.0) containing 0.85% (wt/vol) NaCl. Subsequent quantification of iron ions revealed the coexistence of iron ions and MVs in small density fractions, whereas free ferric iron was not detected in these fractions when subjected to density gradient ultracentrifugation alone (Fig. 2D). The size distributions of the MVs before and after incubation with Fe were similar (Fig. S1C). As several proteins, which we identified in our previous study (12), were present in C. glutamicum MVs produced using -Fe medium, we investigated whether these proteins are involved in the iron-loading activity of MVs. Therefore, we treated the MVs with proteinase K. As presented in the SDS-PAGE data, proteins were only detected before proteinase K treatment of MVs (Fig. 2C), indicating that these proteins are exposed on the MVs surface and degraded by the enzyme. The proteinase K-treated MVs retained iron-loading activity comparable to that of the non-treated MVs, suggesting that the iron-loading activity of C. glutamicum MVs is independent of the MV-associated surface proteins (Fig. 2E).

### Membrane vesicles loaded with ferric iron can promote the growth of Corynebacterium glutamicum in an iron-limited medium.

**FIG 3:**
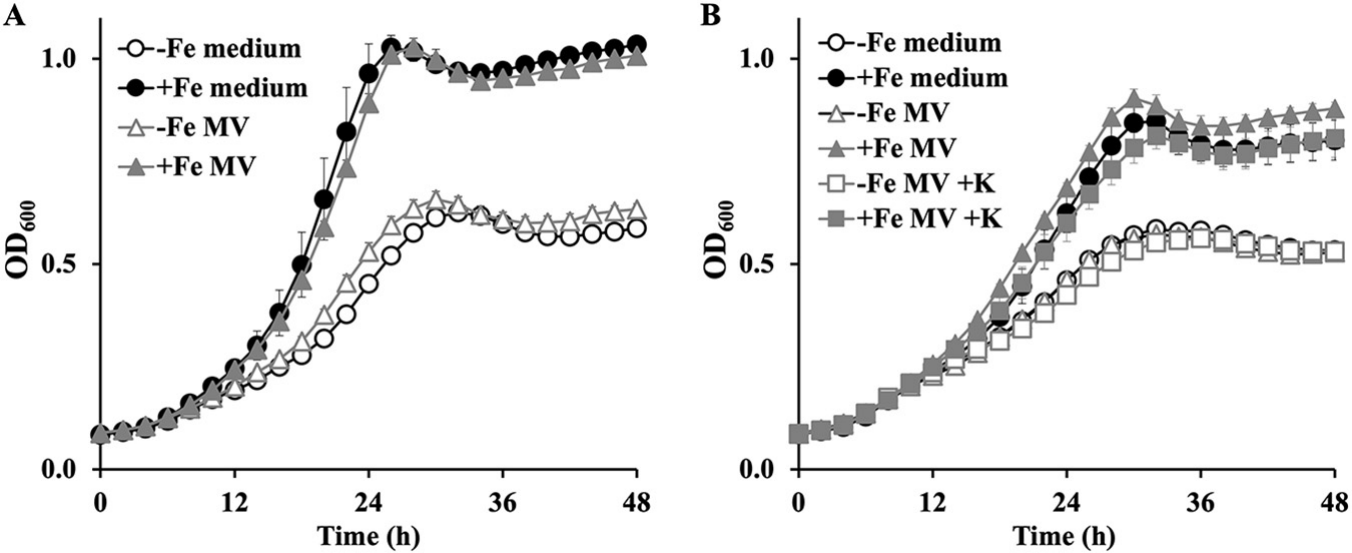
Growth promotion of Corynebacterium glutamicum by membrane vesicles. (A) Growth promotion by iron-loaded MVs. C. glutamicum MVs loaded with ferric iron were added to a -Fe medium, and OD_600_ was recorded at each time point. +Fe MV and -Fe MV indicate iron-loaded MVs and iron-free MVs, respectively. -Fe MV, -Fe MVs-added conditions (open triangle). +Fe MV, +Fe MVs-added conditions (closed triangle). +Fe medium, C. glutamicum WT cultured with supplementing 5 μM FeCl_3_ · 6H_2_O (closed circle). -Fe medium, C. glutamicum WT cultured without iron supplementation (open circle). All values indicated by the bars represent the mean value ± S.D. for five independent cultures. (B) C. glutamicum growth promotion by proteinase K-treated MVs. Proteinase K-treated C. glutamicum MVs were mixed with iron and then purified. The purified MVs were added to a -Fe liquid medium, and OD_600_ was recorded. Circles and triangles correspond to the conditions indicated in panel *A*. -Fe MV +K, -Fe MVs treated with proteinase K (closed square). +Fe MV +K, +Fe MVs treated with proteinase K (open square). All values indicated by the bars represent the mean value ± S.D. for five independent cultures.

Considering C. glutamicum MVs can load iron, we investigated whether these MVs could support the growth of C. glutamicum under iron-limited conditions. We used the -Fe medium, in which the growth of C. glutamicum was promoted by adding FeCl_3_ · 6H_2_O (Fig. 3A). Using the same medium, we examined whether the growth of C. glutamicum could be promoted by the addition of MVs. To prepare iron-loaded MVs (+Fe MVs), MVs were incubated with ferric iron and subjected to density gradient ultracentrifugation to separate the free iron, as described above. The addition of +Fe MVs to the -Fe medium promoted the growth of C. glutamicum comparable to that of FeCl_3_ · 6H_2_O alone (Fig. 3A) and this growth promotion effect was arrested by the further addition of an exogenous iron chelator (Fig. S2). Electron probe microanalyzer (EPMA) analysis also supported our result showing that C. glutamicum cells incubated with +Fe MVs had iron uptake (Fig. S3). MVs that were not incubated with Fe (-Fe MVs) did not promote growth (Fig. 3A). Furthermore, we treated MVs with proteinase K before incubation with ferric iron to determine whether any MV proteins were involved in transporting iron. MVs were purified as described above after incubation with iron. Although most proteins associated with MVs are removed by proteinase K treatment, the treated MVs could still promote the growth of C. glutamicum in the -Fe medium, indicating that the transport of iron through MVs in C. glutamicum is independent of MV-associated surface proteins (Fig. 3B).

### Membrane vesicles are received by Corynebacterium glutamicum cells.

**FIG 4:**
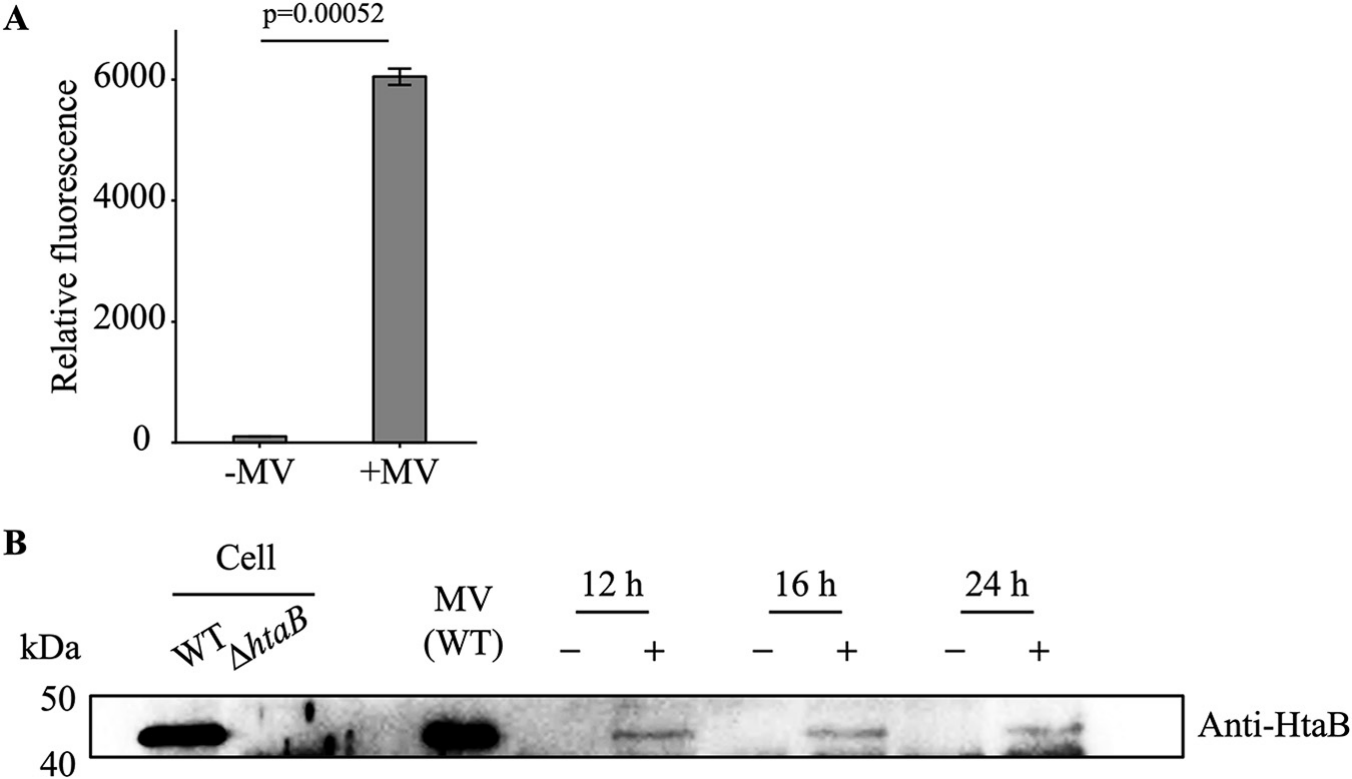
MV delivery to Corynebacterium glutamicum cells. (A) Corynebacterium glutamicum cells that received MVs was detected by a fluorescent dye–based assay. WT MVs stained with FM1-43 FX dye and C. glutamicum Δ*htaB* cells were mixed. The cell fractions were then collected. Relative fluorescence in +MV indicates the relative amount of MVs attached to the cells. -MV shows fluorescence values of non-treated Δ*htaB* cell. All values indicated by the bars represent the mean value ± S.E. for three independent experiments. *P*-values were calculated using a *t* test with Welch's correction. (B) Proteins (each 5 μL per lane) in the total cell lysates or purified MV fractions were separated by SDS-PAGE, followed by Western blotting with anti-HtaB serum. Loading controls are shown in Fig. S3. The collected cell fractions were subjected to Western blotting as described in Materials and Methods.

Considering MVs can transport iron to C. glutamicum cells, we examined whether MVs could be received by C. glutamicum cells. We performed the following fluorescent dye–based assays to detect whether MVs were received. C. glutamicum MVs were purified from the culture as described above and stained with FM1-43 FX, which was then fixed with glutaraldehyde. After washing MVs with 10 mM HEPE-NaOH buffer (pH 8.0) containing 0.85% (wt/vol) NaCl, the stained MVs were added to C. glutamicum cells harvested during the exponential growth phase. The free MVs were removed from the solution by centrifugation and washed with PBS. After mixing the cells with the stained MVs, the normalized fluorescence of the cells significantly increased, indicating that the MVs were attached onto the cell surface that may be further fused with cells (Fig. 4A). Confocal microscope confirmed that the fluorescence signal of FM1-43 FX dye was detected along the cells, and frequent contacts between MVs and the cells were also observed using time-lapse imaging (Movie S1). In addition, we tested the involvement of mycomembrane using a Δ*pks13* mutant that lacks mycomembrane (12). We incubated Δ*pks13* cells with C. glutamicum MVs and harvested the cells for subsequent analyses. We detected MV delivery to Δ*pks13* cells as the fluorescence of the cells increased after incubation of MVs with Δ*pks13* cells (Fig. S4A). Furthermore, MVs from the wild-type strain could promote the growth of the Δ*pks13* mutant in a -Fe medium, suggesting that the mycomembrane is not a major determinant in C. glutamicum cells in uptaking iron from MVs (Fig. S4B).

We further analyzed whether MV cargo was received by the cells using HtaB as a marker, a transmembrane protein abundant in MVs released from C. glutamicum cells (12, 16). HtaB was detected using Western blot analysis of MVs used in this experiment (Fig. 4B). The MVs were incubated with the Δ*htaB* mutant of C. glutamicum and the washed cells were subjected to Western blot analysis. A band corresponding to HtaB was detected in Δ*htaB* cells after incubation with the MVs (Fig. 4B; Fig. S5). In contrast, there was no HtaB signal in the MV-free control sample (Fig. 4B; Fig. S5). We also performed the above-described assays on C. glutamicum Δ*htaB* cells harvested at different growth phases. HtaB was transferred from MVs to cells in all phases, suggesting that this process is growth phase independent.

### Interspecies iron acquisition by Corynebacterium glutamicum membrane vesicles.

**FIG 5:**
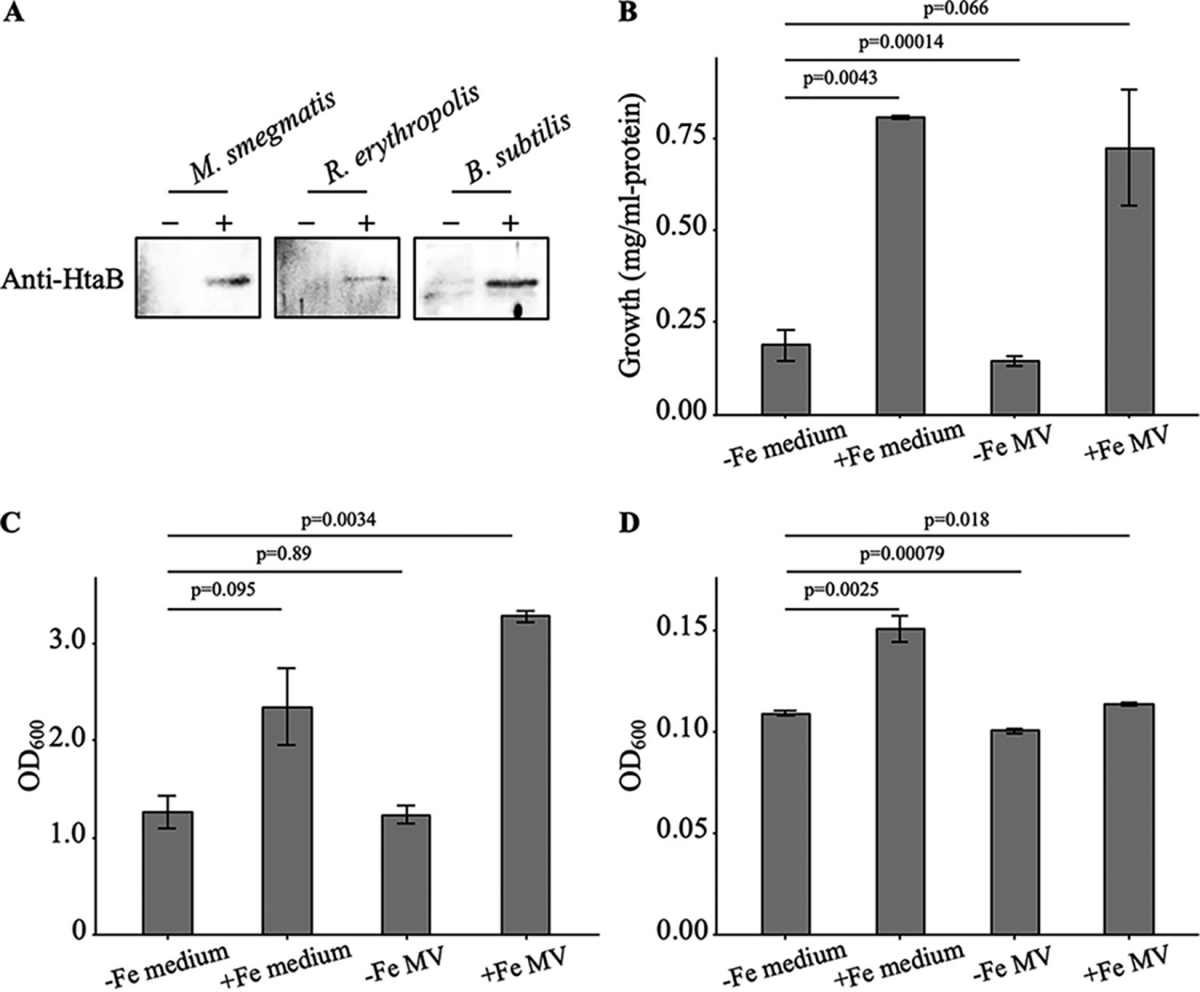
Interspecies uptake of Corynebacterium glutamicum MVs. (A) Corynebacterium glutamicum MVs are received by other bacterial species. C. glutamicum WT MVs and each recipient bacteria (Mycobacterium smegmatis, *R* Rhodococcus erythropolis, and Bacillus subtilis) were mixed, and the cell fractions were then collected. Proteins in the total cell lysates (each 5 μL per lane) were analyzed by SDS-PAGE, followed by Western blotting with rabbit-derived anti-HtaB serum. Loading controls are shown in Fig. S3. Growth of M. smegmatis (B) and R. erythropolis (C) in iron-restricted liquid medium was promoted by iron-loaded MVs of C. glutamicum. The growth promototion effect of C. glutamicum MVs was not observed in B. subtilis (D). C. glutamicum MVs mixed with iron were added to a -Fe medium, and OD_600_ or protein amounts in the cell extracts were measured. -Fe MV, the cultures with the addition of iron-free C. glutamicum MVs. +Fe MV, the cultures with the addition of iron-loaded C. glutamicum MVs. +Fe medium, the cultures with 5 μM FeCl_3_ · 6H_2_O. -Fe medium, the cultures without the addition of FeCl_3_. All values indicated by the bars represent the mean value ± S.E. for three (panels *B* and *C*) or five (panel *D*) independent cultures. *P*-values were calculated using a *t* test with Welch's correction.

Previous studies have implied the species specificity of MV-mediated molecule transport (17, 18). We were interested in whether iron transport mediated by C. glutamicum MVs was species-specific. Purified C. glutamicum MVs were incubated with Gram-positive bacteria, including mycolic acid-containing bacteria (Rhodococcus erythropolis PR4 and Mycobacterium smegmatis MC^2^155) or Bacillus subtilis 168. After washing the cells, the cell extracts were subjected to Western blot analysis using anti-HtaB antiserum, as described above. HtaB was detected in all bacteria tested (Fig. 5A; Fig. S5). Furthermore, we examined whether C. glutamicum MVs can promote the growth of bacteria under iron-limited conditions. The growth of R. erythropolis and M. smegmatis could be promoted to the level of FeCl_3_ · 6H_2_O added alone when +Fe MVs were added, but not with -Fe MVs (Fig. 5B and C). In contrast, the growth of B. subtilis 168 was not promoted by the addition of +Fe, suggesting that there is limited access to MV cargo depending on the species (Fig. 5D).

## DISCUSSION

A siderophore-dependent iron acquisition system has remained unknown in C. glutamicum despite considerable efforts devoted to the identification of siderophore molecules (13–15). In this study, we demonstrated that MVs released by C. glutamicum cells could act as extracellular iron carriers that mediate iron acquisition by this bacterium.

MV-mediated acquisition of iron or heme by mycolic acid-containing bacteria has been reported in Mycobacterium and *Dietzia* species (8, 17). In addition to siderophores containing MV-mediated iron uptake in Mycobacterium tuberculosis (8), in *Dietzia* sp. Dq12-45-1b, a transmembrane heme-binding protein HtaA associated with MVs was proposed to mediate the uptake of extracellular heme *via* direct contact between MVs and cells (17). Our findings on C. glutamicum would be fundamentally different from these previous models of MV-mediated iron acquisition systems in mycolic acid-containing bacteria concerning the iron-loading mechanism of MVs, as it is independent of proteins and typical siderophores associated with MVs (Fig. 2E and 3B).

A possible mechanism of iron capture by C. glutamicum MVs is the chelation of ferric iron by membrane lipids. As reported previously (12), C. glutamicum MVs comprise trehalose corynomycolic acid esters and acidic phospholipids, including phosphatidylglycerol, phosphatidylinositol mannosides, and cardiolipins (Fig. 2B). The β-hydroxy-carbonyl and phosphate moieties of corynomycolic acid esters and phospholipids, respectively, may interact with ferric iron. Since the β-hydroxy-carbonyl moiety can be found within many bacterial ferric iron chelators as iron-binding sites for example, PQS, actinorhodin, and deferoxamine (Fig. S6) (19–21), corynomycolic acid esters, which account for the majority of C. glutamicum MVs lipids (12), may similarly chelate ferric iron. In addition, the acidic phospholipids contained in C. glutamicum MVs may also be involved in iron loading, as suggested in previous studies (22, 23). Alternatively, unidentified iron-binding molecule(s) produced by C. glutamicum may be associated with MVs and account for their iron-loading activity.

Notably, C. glutamicum MVs did not promote the growth of B. subtilis, although B. subtilis was able to receive MVs (Fig. 5). These results suggest that several steps are involved in the uptake of iron from C. glutamicum MVs rather than simple equilibrium diffusion of iron from MVs. The reason why B. subtilis was unable to utilize iron from MVs may be due to the cell wall covering the B. subtilis cell, which inhibits the direct fusion of MVs to the membrane. Notably, C. glutamicum MVs promoted the growth of Δ*pks13* cells lacking mycomembrane under iron-limiting conditions, indicating that the mycomembrane is not a major determinant in receiving MV iron cargo.

In summary, we showed that MVs derived from the mycomembrane of C. glutamicum could chelate ferric iron and mediate iron uptake by this bacterium. These findings would be unique with respect to independence from MV-associated proteins and typical siderophores and the species specificity in the growth-promoting effect of MV-associated iron under iron-restricted conditions. Our findings on C. glutamicum would lead to further clarification of novel strategies for iron acquisition in bacteria and also would give insights in understanding the importance of MV-associated iron in bacterial interactions.

## MATERIALS AND METHODS

### Culture conditions.

All bacteria (Table S1) were precultured in an LB medium. C. glutamicum frozen in glycerol was cultured in LB 1.5% (wt/vol) agar plates at 30°C for 24 h. The colonies were then inoculated into 4 mL of LB medium and incubated at 30°C for 21 h at 190 rpm. Cultured cells were washed twice with synthetic medium without the addition of iron salts (-Fe medium) (80 g/L glucose, 30 g/L [NH_4_]_2_SO_4_, 7.6 g/L Na_2_HPO_4_•7H_2_O, 6 g/L KH_2_PO_4_, 2 g/L NaCl, 1.77 mg/L ZnCl_2_, 0.44 mg/L CuSO_4_•5H_2_O, 5.56 mg/L MnSO_4_•H_2_O, 0.1 mg/L [NH_4_]_6_Mo_7_O_24_•4H_2_O, 0.4 g/L MgSO_4_•7H_2_O, 84 mg/L CaCl_2_, 500 μg/L thiamine-HCl, 200 μg/L biotin, the pH was adjusted to 7.2, using KOH) and then incubated into 100 mL of -Fe medium at 1.5% (vol/vol). C. glutamicum was then incubated at 30°C for 27 h at 150 rpm (late exponential growth phase).

For the Δ*pks13* mutant of C. glutamicum, biotin was added to the LB medium at 200 μg/L and cultured at 30°C for 72 h for subsequent experiments.

Mycobacterium smegmatis was cultured in LB 1.5% (wt/vol) agar plates at 37°C for 3 days. Colonies were then inoculated into 4 mL of LB medium and incubated at 37°C for 3 days at 190 rpm for preculture. M. smegmatis was inoculated into 100 mL of a minimum medium comprising 0.7% (vol/vol) glycerol, 1 g KH_2_PO_4_, 2.5 g Na_2_HPO_4_, 0.5 g asparagine, 0.5 g MgSO_4_•7H_2_O, 0.5 mg CaCl_2_, 0.1 mg ZnSO_4,_ and 200 μg biotin per 1 L. The pH was adjusted to 7.0, using KOH.

Rhodococcus erythropolis was cultured in LB 1.5% (wt/vol) agar plates at 30°C for 24 h. The colonies were then inoculated into 4 mL of LB medium and incubated at 30°C for 24 h at 190 rpm precultures. The preculture was then inoculated into the same synthetic medium used for C. glutamicum culture.

Bacillus subtilis was cultured in LB 1.5% agar plates at 37°C for 12 h. Colonies were then inoculated into 4 mL of LB medium and incubated at 30°C for 16 h at 190 rpm for preculture. The preculture was then inoculated into iron-limited MC medium comprising 1 g casamino acid, 3 mL 1 M MgSO_4_, 50 mg tryptophan, 20 g glucose, 2 g glutamate-Na, 13.6 g, KH_2_PO_4_ per 1 L. The pH was adjusted to 7.0, using NaOH.

### In-frame deletion of htaB.

The Δ*htaB* mutant of C. glutamicum was constructed as follows: The flanking regions of *htaB* were amplified by PCR using the primers listed in Table S2. These amplified sequences were fused with pK18mobsacB (digested with EcoRI and HindIII) using In-Fusion. The resultant plasmid was replicated in E. coli DH5α and introduced into C. glutamicum by electroporation and subsequent heat shock. The transformants were selected using kanamycin. Finally, the in-frame deletion mutant of *htaB* was isolated from a sucrose-containing solid medium and confirmed by PCR.

### Membrane vesicles purification and quantification.

The MVs were isolated and quantified as the culture was centrifuged at 4°C for 10 min at 7000 × *g*, and the bacteria were pelleted. After centrifugation, the supernatant was filtered through a 0.45 μm pore size PVDF filter (Merck Millipore, Germany) to remove bacteria. The supernatant was ultracentrifuged at 4°C for 1 h at 150 k × *g* and used as crude MVs. To further purify MVs, the pellet was resuspended in a buffer containing 45% (wt/vol) iodixanol (Optiprep, AXIS-SHIELC, Dundee, Scotland) in 400 μL of 10 mM HEPES (pH 8.0-NaOH) + 0.85% (wt/vol) NaCl solution. This solution was placed at the bottom of a 4 mL tube; 400 μL of 40, 35, 30, 25, 20, and 15% iodixanol solution and 200 μL of 10% iodixanol solution layered on the top of the suspended solution to make density gradient in the tube. The tubes were ultracentrifuged at 4°C for 3 h at 100 k × *g*. After ultracentrifugation, a fraction (400 to 800 μL) containing purified MVs that appeared as a band in the upper center of the tube was collected and washed in 10 mM HEPES (pH 8.0-NaOH) + 0.85% (wt/vol) NaCl solution using ultracentrifugation. The pellet was resuspended in the same buffer and used for further analyses. MVs were quantified using the fluorescent dye FM1-43 FX (excitation/emission wavelength: 479 nm/598 nm).

### SDS-polyacrylamide gel electrophoresis (SDS-PAGE).

Protein quantification of MVs was performed colorimetrically using a bicinchoninic acid (BCA) protein assay kit (Thermo Fisher Scientific) in a plate reader (Abs_570_). Each sample was mixed with 2 × SDS-PAGE sample buffer (0.5 M Tris (pH 6.8), glycerol, 4% (wt/vol) SDS, 5% (vol/vol) 2-merceptoethanol, 0.2% (wt/vol) bromophenol blue) and heat treated using a heat block at 95°C for 5 min. Protein (5 μg per lane) was added to 12% polyacrylamide gel (running gel: 3.3 mL MilliQ water, 4 mL 30% [wt/vol] acrylamide, 2.5 mL 3.0 M Tris-HCl [pH 8.8], 100 μL 10% [wt/vol] SDS, 100 μL 10% [wt/vol] APS, 4 μL TEMED, stacking gel: 2.7 mL MilliQ water, 670 μL 30% [wt/vol] acrylamide, 500 μL 1.0 M Tris-HCl [pH 6.8], 40 μL 10% [wt/vol] SDS, 40 μL 10% [wt/vol] APS, 8 μL TEMED). After electrophoresis, the gel was stained with Coomassie brilliant blue.

### Nanoparticle tracking analysis.

The size distribution and particle numbers of the MVs were measured by the nanotracking method using a Nanosight NS300 (Nanosight Ltd., Malvern, United Kingdom). Purified MVs samples were used and diluted with 10 mM HEPES (pH 8.0-NaOH) + 0.85% (wt/vol) NaCl solution, as appropriate. The flow rate was set to 100 for the analysis, and the average of three 60-s video recordings was used.

### Electron microscopic observation of membrane vesicles.

Transmission electron microscopy (Hitachi High-Technologies, H-7650), TEM grid (Cu400CN, ALLIANCE Biosystems, Osaka, Japan), and EM Stainer (Nissin EM) for negative staining were used for observation. The grids were placed on top of a droplet of the appropriately diluted MVs sample and allowed to stand for 30 s. The grid was then transferred onto a 50 μL droplet of MilliQ water for washing and allowed to stand for 10 s. The grid was then transferred onto a 10 μL droplet of EM Stainer and was allowed to stand still for 30 s. An excess staining agent was absorbed with a prowipe and allowed to dry naturally. Completely dried samples were observed under a transmission electron microscope.

### CAS assay.

The iron-loading activity of MVs was determined by CAS assay (24), which is widely used to detect siderophore production: Solution I (60.5 mg chrom azurol S, 50 mL MilliQ water), Solution II (72.9 mg HDTMA, 40 mL MilliQ water), Solution III (2.7 mg FeCl_3_ · 6H_2_O, 10 mM HCl) was prepared. Solution III (10 mL) was then slowly added to the solution I, followed by solution II. Subsequently, 30.24 g PIPES, 2g tryptone, 1 g yeast extract, and 4 g NaCl were dissolved in 900 mL DW, and pH was adjusted to 6.8 with NaOH. This PIPES-LB solution was sterilized by autoclaving after addition of 12 g agar. The plate was prepared by mixing the solution and PIPES-LB containing agar. MV solution was sterilized on a 0.45 μm filter, and 5 μL drops were added. For the CAS assay, C. glutamicum cultured in liquid LB medium at 30°C overnight was plated on LB agar medium and incubated at 30°C for 24 h to form colonies. They were then layered with a mixture of autoclaved Solutions I, II, and III and agar. The colonies were incubated at 30°C for 3 days to check for clear zones.

### Incubation of ferric iron with MVs.

The MVs were isolated from the -Fe medium. The crude MVs were mixed with FeCl_3_ · 6H_2_O (Fin. conc. 150 μM) and allowed to stand at 30°C for 2 h. After ultracentrifugation at 4°C for 1 h at 150 k × *g*, MVs were purified as described above using density gradient ultracentrifugation at 4°C for 1 h at 150 k × *g*. After ultracentrifugation, 500 μL was fractionated from the top of the tube. All fractions were washed in 10 mM HEPES-NaOH (pH 8.0) + 0.85% NaCl solution. The pellet was resuspended in the same buffer and used for further analyses.

### Quantification of iron ions.

The concentration of iron ions (Fe^2+^ and Fe^3+^) in the MV solution was determined colorimetrically using the ferrozine method. The Metalloassay Iron Assay LS (MG Metallogenics, Chiba, Japan) was used for quantification. The sample (40 μL) was mixed with 200 μL of R-A buffer provided in the kit in a 96-well plate (IWAKI) and allowed to stand for 5 min. Abs_570_ was measured in a microplate reader using the sample blank as a control, and Abs_A_ was determined. Subsequently, 8 μL of the chromogenic reagent R-R Chelate color was added, mixed, and allowed to stand for 5 min, after which the Abs_570_ was measured using a reagent blank as a control and designated Abs_B_. The concentration of iron ions in the solution was determined by comparing the Abs_B_-Abs_A_ obtained from each sample with that of the iron standard solution supplied with the kit.

### Lipid analysis of membrane vesicles.

Lipid analysis of MVs was performed based on previous reports (6). MV lipids were extracted by adding chloroform:methanol (2:1) solvent to lyophilized MVs and stirring at room temperature for 1 h at 1400 rpm. The extracted lipids were separated using thin-layer chromatography as follows: Samples were spotted onto a silica gel plate and separated using chloroform:methanol: H_2_O = 65:25:4 (vol/vol). The silica gel plate was dried and stained with 0.05% (wt/vol) Primulin dissolved in 80% (vol/vol) acetone. The fluorescence of the labeled lipids was detected at 365 nm.

### Growth measurement.

The MVs were recovered from C. glutamicum grown in a -Fe medium and purified after incubation with iron. C. glutamicum was grown in LB medium at 30°C for 18 h at 190 rpm, washed twice with -Fe medium, inoculated with 1% of the culture medium containing the iron source, and grown in a 96-well-plate at 30°C for 48 h. The growth curve was recorded by measuring the OD_600_ using Bio tek cytation 5 (Agilent Technologies, CA, USA). Culture mediums to which MVs were added were sterilized by filtering through a 0.45 μm pore size filter. C. glutamicum Δ*pks13* was grown in 200 μg/mL biotin LB medium at 30°C for 3 d at 190 rpm, washed twice with 4 mL of -Fe medium, inoculated with 1% in the same -Fe medium with and without different iron sources, and grown at 30°C for 5 d. The growth was recorded by measuring the amount of protein. M. smegmatis was grown in LB medium at 37°C for 3 d at 190 rpm, washed twice with 4 mL of -Fe medium, inoculated with 1% in the same -Fe medium with and without different iron sources, and grown at 37°C for 5 d. Growth was recorded by measuring the amount of protein. R. erythropolis was grown in LB medium at 30°C for 24 h at 190 rpm, washed twice with 4 mL of -Fe medium, inoculated at 1% in the same -Fe medium with and without different iron sources, and grown at 30°C for 72 h. Growth was recorded by measuring OD_600_. B. subtilis was grown in LB medium at 30°C for 16 h at 190 rpm, washed twice with 4 mL of -Fe medium, inoculated with 1% in the same -Fe medium with and without different iron sources, and grown in 96-well-plate at 30°C for 12 h. The growth curve was recorded by measuring the OD_600_ using Bio tek cytation 5 (Agilent Technologies, CA, USA).

### Fluorescence-based detection of MV-cell attachment.

The MVs were recovered from -Fe media and purified. MV solution (1 × 10^11^ particles/mL) was stained with 2.5 μg/mL FM1-43 FX and fixed with 4% (vol/vol) glutaraldehyde solution. Afterward, the solution was washed using ultracentrifugation at 4°C for 1 h at 150 k × *g* with 10 mM HEPES pH 8.0 + 0.85% (wt/vol) NaCl buffer. C. glutamicum Δ*htaB* cells were cultured in LB medium at 30°C and 190 rpm and washed twice with PBS. C. glutamicum Δ*pks13* cells were cultured in LB medium with 200 μg/mL biotin at 30°C for 18 h at 190 rpm and washed twice with PBS. The MVs solution was sterilized using a 0.45 μm filter and added to the recipient cells in PBS. The ratio of MV to recipient cells was adjusted to 1,000:1. After 1 h of incubation at 30°C, the cells were centrifuged at 4°C for 5 min at 3,000 × *g*, and fractionated into supernatant and cell (pellet) fractions. The relative fluorescence was quantified (excitation/fluorescence wavelength: 479 nm/598 nm). The stained cells were observed using an Olympus SpinSR10 microscope equipped with a UAPON 100XOTIRF lens.

### Western blotting.

The MVs were recovered from -Fe media and purified. C. glutamicum cells were cultured in LB medium at 30°C for 18 h at 190 rpm and washed twice with PBS. *M. smegatis* cells were cultured in LB medium at 37°C for 3 d at 190 rpm, R. erythropolis cells were cultured in LB medium at 30°C for 24 h at 190 rpm, B. subtilis cells were cultured in LB medium at 37°C for 16 h at 190 rpm and washed twice with PBS. The MVs solution was sterilized using a 0.45 μm filter and added to the recipient cells in PBS. The ratio of MV to recipient cells was adjusted to 1,000:1. After 6 h at 30°C and 1400 rpm, the cells were centrifuged at 4°C for 5 min at 3,000 × *g* and pelleted. The cell pellets were resuspended in 200 μL of PBS with a protease inhibitor cocktail (Roche Diagnostic, Basel, Switzerland) and disrupted with 0.1 mm silica spheres (MP Biomedicals, CA, USA) for 45 s. The cell extracts were subjected to SDS-PAGE as described above. The gel was transferred to a PVDF membrane and blocked with 5% (wt/vol) skim milk in TBS containing 0.05% (vol/vol) Tween 20. The membrane was reacted with anti-HtaB serum (1:500 dilution), which was raised in a rabbit using the HtaB peptide (NH2-C+PETSVDPEKPGDDN-COOH), and then with HRP-conjugated anti-rabbit IgG antibody (1:5000 dilution; FUJIFILM Wako, Chuo, Osaka, Japan). Signals were detected using Immunostar LD (FUJIFILM Wako).

### Electron probe micro analyzer (EPMA).

The MVs were recovered from a -Fe medium and purified by mixing with FeCl_3_ · 6H_2_O (Fin. conc. 1 mM) as described above. The recipient cells were grown in LB medium at 30°C overnight at 190 rpm and washed twice with PBS. The cells were mixed at 30°C for 30 min at 1400 rpm and then centrifuged at 4°C for 5 min at 3000 × *g* to remove excess MVs remaining in the supernatant. Cells were fixed with 2.5% (vol/vol) glutaraldehyde, dropped onto carbon, and dried for analysis.

### Data availability.

Data will be made available upon request to the corresponding author.
