# Supplementary material for: Iron Delivery through Membrane Vesicles in Corynebacterium glutamicum
Source: Microbiol Spectr. 2023 May 8;11(3):e01222-23. doi: 10.1128/spectrum.01222-23 (PMC10269601; doi:10.1128/spectrum.01222-23)
Supplement: Supplemental file 2 — Supplemental material. Download spectrum.01222-23-s0001.pdf, PDF file, 2.6 MB [file spectrum.01222-23-s0001.pdf]

Supplemental Material

Iron delivery through membrane vesicles in *Corynebacterium glutamicum*

Authors:
Kayuki Kawashima[1†], Toshiki Nagakubo[2,3†], Nobuhiko Nomura[2,3], Masanori Toyofuku[2,3]*

Affiliations:
[1]Graduate School of Life and Environmental Sciences, University of Tsukuba, 1-1-1 Tennodai, Tsukuba, Ibaraki, Japan.
[2]Faculty of Life and Environmental Sciences, University of Tsukuba, 1-1-1 Tennodai, Tsukuba, Ibaraki, Japan.
[3]Microbiology Research Center for Sustainability (MiCS), University of Tsukuba, 1-1-1 Tennodai, Tsukuba, Ibaraki, Japan.

[†]Equal contribution: Kayuki Kawashima and Toshiki Nagakubo equally contributed to this study.

*Corresponding author: Masanori Toyofuku
Email: toyofuku.masanori.gf@u.tsukuba.ac.jp

Content:
Supplemental Figures 1-6
Supplemental Tables 1 and 2
Supplemental Movie

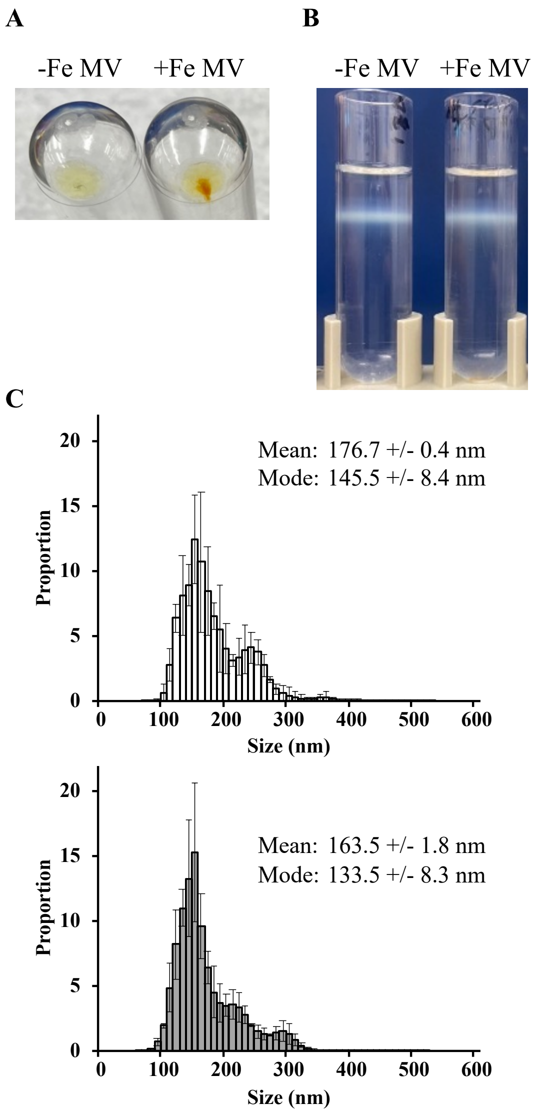

Supplemental Figure 1. MVs mixed with ferric iron.

(A) Pellets of MV mixed with iron after ultracentrifugation are shown. Non-treated MVs (-Fe MV, left) and MVs incubated with ferric iron (+Fe MV, right) were ultracentrifuged. (B) Representative results of density gradient centrifugation of MVs are shown. Iron-free MVs (-Fe MV, left) and iron-loaded MVs (+Fe MV, right) were subjected to density-gradient ultracentrifugation. White bands correspond to the MVs. (C) Particle size distribution of the purified MVs mixed with ferric iron is shown. Non-treated MVs (-Fe MV, top) and iron-loaded MVs (+Fe MV, bottom) were analyzed.

A

B

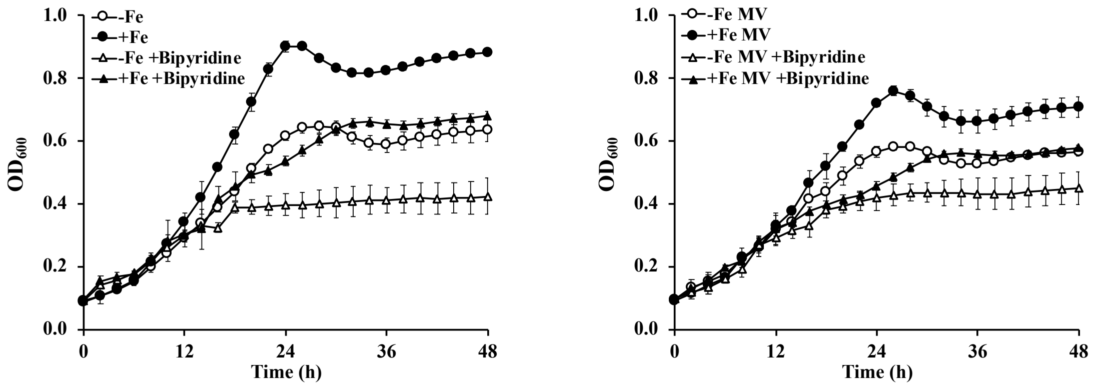

Supplemental Figure 2. Growth arrest of *Corynebacterium glutamicum* by an iron chelator.

(A) Growth promoted by $FeCl_3 \cdot 6H_2O$ was arrested by an iron chelator (2,2′-bipyridine). 2,2′-Bipyridine was added to a -Fe medium at 10 µM, and OD_{600} was recorded at each time point. +Fe medium, *C. glutamicum* WT cultured with 5 µM $FeCl_3 \cdot 6H_2O$ supplied (closed circle). -Fe medium, *C. glutamicum* WT cultured without iron supplied (open circle). +Fe +Bipyridine, 2,2′-bipyridine was added to +Fe medium (closed triangle). -Fe +Bipyridine, 2,2′-bipyridine was added to -Fe medium (open triangle). All values indicated by the bars represent the mean value ± S.D. of three independent cultures. (B) Growth promotion by iron-loaded MVs was arrested by an iron chelator (2,2′-bipyridine). *C. glutamicum* MVs loaded with ferric iron were added to a -Fe medium with 2,2′-bipyridine, and OD_{600} was recorded at each time point. 2,2′-bipyridine was added to the -Fe medium at 10 µM. +Fe MV and -Fe MV indicate iron-loaded MVs and iron-free MVs, respectively. -Fe MV, -Fe MVs-added conditions (open circle). +Fe MV, +Fe MVs-added conditions (closed circle). -Fe MV +Bipyridine, -Fe MVs-added with 2,2′-bipyridine (open triangle). +Fe MV +Bipyridine, +Fe MVs-added with 2,2′-bipyridine (closed triangle). All values indicated by the bars represent the mean value ± S.D. for three independent cultures.

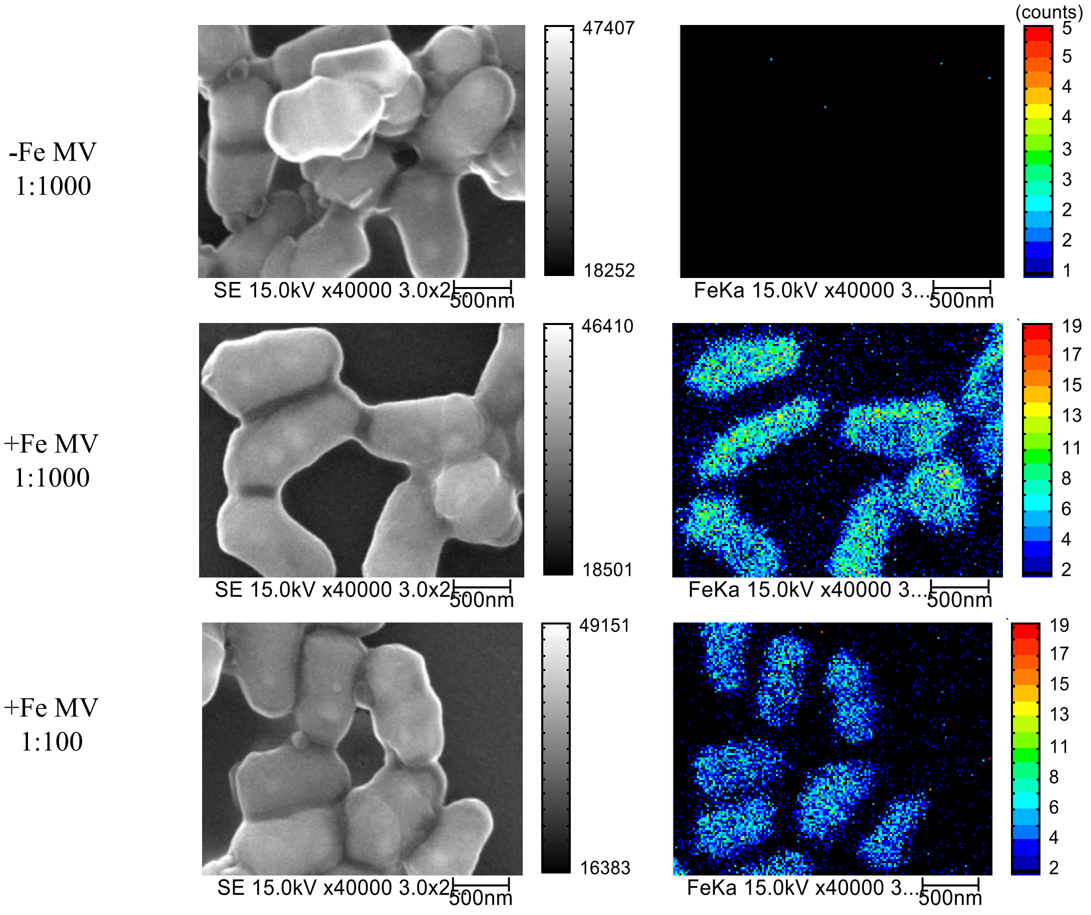

Supplemental Figure 3. Iron uptake from iron-loaded MVs to cells.

Scanning Electron Microscope (SEM) observation and electron probe micro analyzer (EPMA) detection of iron from *C. glutamicum* cells. MVs isolated from -Fe medium were purified by density gradient ultracentrifugation after mixing with $FeCl_3$ (final concentration 1 mM). Iron-loaded MVs (+Fe MV, middle and bottom) and iron-free MVs (-Fe MV, top) were added at a ratio of 100 or 1000 particles per cell. Scale bar 500 nm.

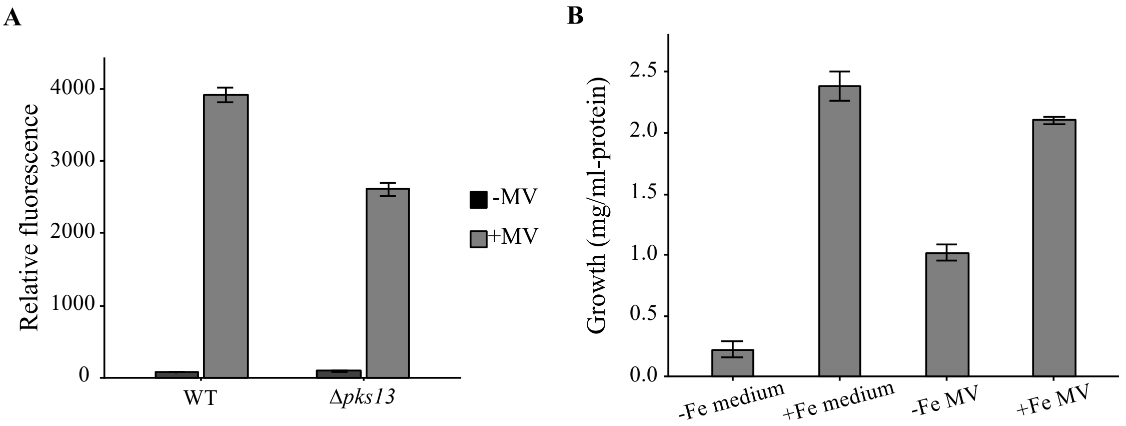

Supplemental Figure 4. MV-mediated iron delivery in mycolic acid-deficient *C. glutamicum* cells.

(A) *C. glutamicum* MVs are received by a Δ*pks13* mutant lacking a key mycolic acid synthesizing enzyme. WT MVs stained with FM 1-43 FX dye and *C. glutacmium* WT or Δ*pks13* cells were mixed and then the cell fractions were collected by centrifugation. Relative fluorescence indicates the relative amount of MVs attached to the cells. All values indicated by the bars represent the mean value ± S.E. for three independent experiments. (B) *C. glutamicum* Δ*pks13* growth in -Fe medium was promoted by *C. glutamicum* iron-loaded MVs. *C. glutacmium* MVs loaded with iron were added to -Fe medium and OD_{600} was measured. +Fe MV and -Fe MV indicate iron-loaded MVs and iron-free MVs, respectively. -Fe MV, the cultures with the addition of iron-free *C. glutamicum* MVs. +Fe MV, the cultures with the addition of iron-loaded *C. glutamicum* MVs. +Fe medium, *C. glutamicum* Δ*pks13* cultured with supplementating 5 μM $FeCl_3 \cdot 6H_2O$ (PC, closed circle). -Fe medium, *C. glutamicum* Δ*pks13* cultured without iron supplementation (NC, open circle). All values indicated by bars represent the mean ± S.E. of three independent cultures.

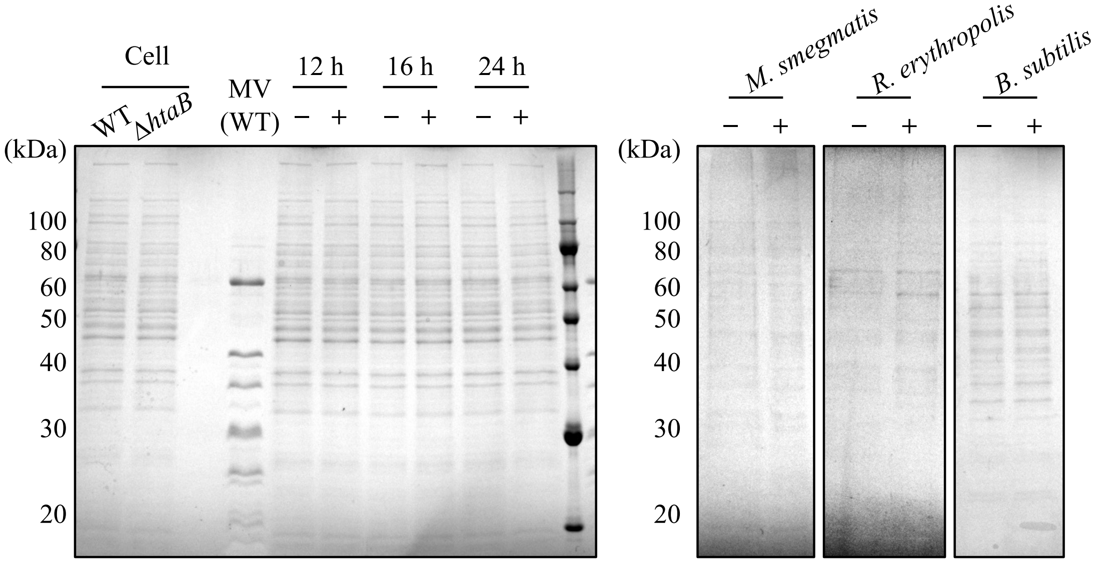

Supplemental Figure 5. SDS-PAGE of cells and MVs

Proteins in the total cell lysates (5 μl per lane) and purified MV fractions (5 μg per lane) were analyzed by SDS-PAGE, followed by Coomassie Brilliant Blue (CBB) staining. The CBB-stained samples serve as the loading controls for western blotting in Figs. 4B (left) and 5A (right).

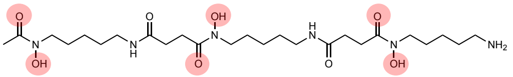

Deferoxamine

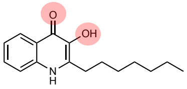

PQS

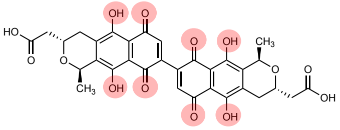

Actinorhodin

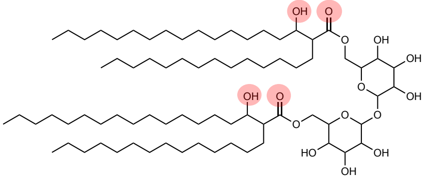

Trehalose di-corynomycolic acid

Supplemental Figure 6. Comparison of chemical structures between corynomycolic acid and bacterial ferric chelators.

Ferric chelators produced by bacteria and trehalose di-corynomycolic acid are shown. Red circles indicate β-hydroxy-carbonyl moieties which are predicted to be involved in chelation of ferric iron.

Supplemental Table 1. Bacterial strains and a plasmid used in this study.

Strains	Characteristics	Reference or source
Corynebacterium glutamicum ATCC13032 (NBRC12168)	Wild-type strain	NBRC
Corynebacterium glutamicum Δ*htaB*	Δ*Cgl0392* mutant of wild-type strain	This study
Corynebacterium glutamicum Δ*pks13*	Δ*pks13* mutant of wild-type strain	(1)
Mycobacterium smegmatis MC^2155	Wild-type strain	(2)
Rhodococcus erythropolis PR4	Wild-type strain	(3)
Bacillus subtiis 168	Wild-type strain	(4)
Escherichia coli DH5α	F- , Φ80d*lacZ*ΔM15, Δ(*lacZYA-argF*)U169, *deoR*, *recA*1, *endA*1, *hsdR*17(r_K^- , m_K^+), *phoA*, *supE*44, λ- , *thi*1, *gyrA*96, *relA*1	TAKARA Bio Inc.

plasmid	Characteristics	Reference or source
pK18mobSacB	A plasmid for gene deletion in *C. glutamicum*, KanR	(5)

Supplemental Table 2. Primers used in this study.

Names and nucleotide sequences of primers used in this study are shown. Restriction sites are underlined.

Primer	Sequences
Cgl0392_FR1_Fd	TATGACCATGATTAC<u>GAATT</u>CATCCGATATGGCCAGGGTGC
Cgl0392_FR1_Rv	TCGATGCCACCGTGGTATTTGTATG
Cgl0392_FR2_Fd	AAATACCACGGTGGCATCGA TGACAACAAGGATGACGGAAGCA
Cgl0392_FR2_Rv	ACGACGGCCAGTGCC<u>AAGCT</u>CCACCTCTACGGTGACAGCGA

Supplemental Movie 1. ***Corynebacterium glutamicum*** **MVs interacting with cells.**
WT MVs fixed with FM 1-43 FX dye and *C. glutamicum* Δ*htaB* cells were mixed. Cell fractions were then collected. Stained cells were observed under a confocal microscope Olympus SpinSR10 mounted with a UAPON 100XOTIRF lens. Times are shown in minutes and seconds.

References

1. Nagakubo T, Tahara YO, Miyata M, Nomura N, Toyofuku M. 2021. Mycolic acid-containing bacteria trigger distinct types of membrane vesicles through different routes. iScience 24:102015.
2. Mohan A, Padiadpu J, Baloni P, Chandra N. 2015. Complete genome sequences of a *Mycobacterium smegmatis* laboratory strain (MC^2 155) and isoniazid-resistant (4XR1/R2) mutant strains. Genome Announc. 3:4-5.
3. Komukai-Nakamura S, Sugiura K, Yamauchi-Inomata Y, Toki H, Venkateswaran K, Yamamoto S, Tanaka H, Haruyama S. 1996. Construction of bacterial consortia that degrade Arabian light crude oil. J Ferment Bioeng. 82:570-574.
4. Bohin JP, Rigomier D, Schaeffer P. 1976. Ethanol sensitivity of sporulation in *Bacillus subtilis*: a new tool for the analysis of the sporulation process. J Bacteriol. 127:934-940.
5. Schäfer A, Tauch A, Jsger W, Kalinowski J, Thierbachb G, Piihler A. 1994. Small mobilizable multi-purpose cloning vectors derived from the *Escherichia coli* plasmids pK18 and pK19: selection of defined deletions in the chromosome of *Corynebacterium glutumicum*. Gene. 145:69-73.
